# Sleep Psychologically Considered, with Reference to Sensation and Memory

**Published:** 1850-09

**Authors:** 


*Sleep psychologically considered, with reference to Sensation and Memory.* By BLANCHARD FOSGATE, M. D., Physician to the New York State Prison, at Auburn. Published by George P. Putnam, New York. 1850.

The author of this volume is a physician of eminence, and well known as a scholar. He has contributed many excellent articles to the American

Journal of Medical Sciences, at Philadelphia, and to other medical and literary journals. In the present work he has well sustained his reputation, and has made a valuable contribution to American literature.

The subjects discussed are Nervous and Mental Action, Sleep, Mesmerism, Somnambulism, Incubus, Trance, and Catalepsy. Upon each of these separate topics he is original, ingenious, and often conclusive. He is, however, a convert to both Phrenology and Mesmerism, and he has interwoven enough "facts" from each into his arguments and speculations, to create occasionally a doubt as to the stability of the structure which in part depends upon them. Aside of this, however, the book has so many and such unequivocal merits as to commend itself to every intelligent or metaphysically inclined reader. F. H. H.

